# Child and adolescent health in Europe: Towards meeting the 2030 agenda

**DOI:** 10.7189/jogh.13.04011

**Published:** 2023-01-20

**Authors:** Minhye Park, Sanja Budisavljević, Aixa Y Alemán-Díaz, Susanne Carai, Katharina Schwarz, Aigul Kuttumuratova, Lei B Jobe, Vivien Hülsen, Yae Eun Lee, Eileen Scott, Ross Whitehead, Martin W Weber

**Affiliations:** 1 WHO Regional Office for Europe, Copenhagen, Denmark; 2 University of St Andrews, St Andrews, Scotland, UK; 3 Copenhagen Business School, Copenhagen, Denmark; 4 Universitat Witten/Herdecke, Witten, Germany; 5 Public Health Scotland, Edinburgh, Scotland, UK; 6 Public Health Scotland, Glasgow, Scotland, UK

## Abstract

**Background:**

Childhood and adolescence are critical stages for a healthy life. To support countries in promoting health and development and improving health care for this age group, the WHO Regional Office for Europe developed the European strategy for child and adolescent health 2015-2020, which was adopted by all countries. This paper reports progress in the strategy’s implementation until 2020.

**Methods:**

A survey was sent to all ministries of health of the 53 Member States of the WHO European Region. Responses were received from 45 Member States. Results are presented in this paper.

**Results:**

The European Region made overall progress in recent years, but increasing levels of overweight and obesity among children, adolescent mental health and low breastfeeding rates are recognized as key national challenges. Although forty-one countries adopted a national child and adolescent health strategy, only eight countries involve children in their review, development and implementation stages. Two-thirds of countries have a strategy for health-promoting schools and a school curriculum for health education. One-third of countries do not have legislation against marketing of unhealthy foods and beverages to children. Most countries reported routine assessment for developmental difficulties in children, but less than a quarter collected and reported data on children who are developmentally on track. There are major gaps in data collection for migrant children. Hospitalization rates for young children vary five-fold across the region, indicating over-hospitalization and access problems in some countries. Only ten countries allow minors access to health care without parental consent based on their maturity and only eleven countries allow school nurses to dispense contraceptives to adolescents without a doctor’s prescription.

**Conclusions:**

This paper shows the progress in child and adolescent health made by countries in Europe until 2020 and key areas where additional work is needed to move the 2030 agenda forward. The survey was undertaken before the COVID-19 pandemic and the war in Ukraine. Both will likely exacerbate many of the observed problems and potentially reverse some gains reported. A renewed commitment is needed.

The child and adolescent years are crucial for establishing a healthy foundation in life. The last decade saw a surge in commitment to child and adolescent health (CAH) both in Europe and globally. In 2014, the World Health Organization (WHO) European region adopted its second strategy, “Investing in children: child and adolescent health strategy for Europe 2015-2020” [1] which built on a previous strategy launched in 2005 [2]. All 53 Member States of the WHO European region endorsed its vision that children and adolescents in Europe should realize their full potential for health, development, and well-being and committed to reducing child mortality and improving the quality of care for children and adolescents.

However, the coronavirus disease 2019 (COVID-19) and the war in Ukraine have seriously impacted the lives of children and adolescents in the region. The pandemic especially revealed weak health, social, and financial systems failing to protect children and adolescents. Preventive measures such as immunization and well-child visits were often halted and curative care became more difficult to access due to lack of service provision or fear of accessing services when needed [3]. The war in Ukraine exacerbated the situation for many children and their families and further disrupted the progress towards reaching the Sustainable Development Goals (SDG), reversing decades of action to realize children’s health and rights. It is therefore crucially important to review what the European region has achieved in terms of child and adolescent health, to understand the needs, build on the achievements, address shortcomings, and future-proof the next European roadmap toward the 2030 Agenda for this age group (see Appendix S1 in **Online Supplementary Document**).

Monitoring progress within the countries has been central to implementing the strategies. Progress was monitored by collecting data through surveys which included areas where countries’ efforts contribute towards achieving the vision set out in the European strategy 2015-2020. Countries responded to the first survey and reported to WHO in 2017, which results were published in a report and article [4,5]. Reaching the end year of the strategy in 2020, countries were surveyed again to report on further progress.

This article assesses the situation of child and adolescent health in the region compared to the previous situation report from 2017 [4], summarizes the findings of the progress from the survey, and highlights possible areas for action within and across countries. As countries reported back before the COVID-19 pandemic started, the findings do not reflect the effect of the pandemic. The overview of findings is presented in a WHO report by summary tables at the end of each health area where all of the data mentioned in the article and further relevant information are shown at a glance [6]. The findings can inform future international and national efforts to ensure that all children and adolescents have a fair shot at better and more equitable health outcomes along their life course.

## METHODS

### Survey design and study cohort

The European strategy 2015-2020 provided the basis for the indicators used to assess progress of child and adolescent health in the region for both the 2017 and the 2020 survey. The surveys collected information directly from 53 Member States in the WHO European region on areas where data were not publicly available and where more in-depth information was needed on available data (see Appendix S3 in the **Online Supplementary Document** for the 53 Member States).

The 2020 survey was created in three steps. First, the survey sent out in 2017 was reviewed to determine which questions would be asked again and new areas that needed to be included. Experts in other WHO programmes, and WHO Collaborating Centres provided suggestions for new items to explore with Member States. The determined questions and items were those that had previously not been asked by other institutions or agencies and others that had been asked before, but lacked information in corresponding institution or agency databases. Country profiles and materials developed as part of a possible new regional strategy were reviewed to pre-fill the repeated questions from the 2017 survey in the next step. Lastly, all questions were reviewed and made available in English and Russian. Overall, 66 questions were included in the 2020 survey (see Appendix S2 in the **Online Supplementary Document****)**.

### Data collection and statistical analysis

The questionnaire was set up using an online survey tool (LimeSurvey GmbH, Germany) and was sent out to the ministries of health in the WHO European Region in November 2019 via email participation link. Forty-five out of 53 countries responded to the survey during the period of November 2019 to January 2020. Non-responding countries were sent reminders, some of the non-responders stated they did not have the capacity or time. Submitted responses were reviewed for missing answers, or discrepancies between responses to quantitative and qualitative questions. Descriptive analysis was conducted via the statistical computing software R and Microsoft Excel. Results are presented through maps, bar charts, and box plots created through the geospatial software ArcGIS Pro (version 10.7), Microsoft Excel, and statistical computing software R. More details of the survey methodology and results of the survey by country are included in a WHO report [6].

## RESULTS

### Problem areas of child and adolescent health identified by countries

**Figure 1:**
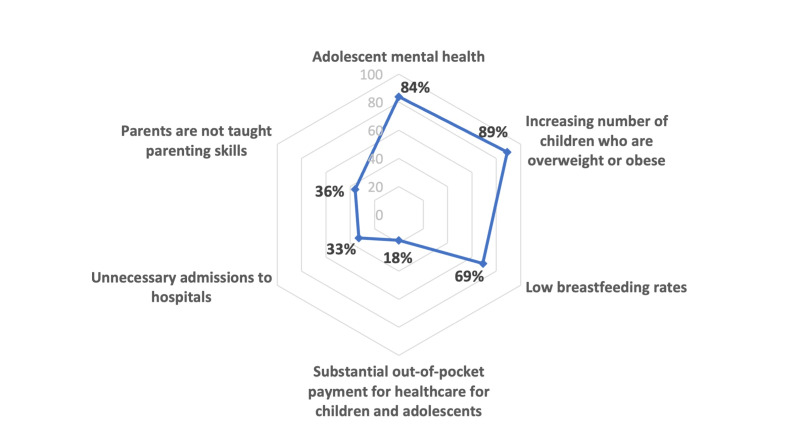
Spider graph of responses from 45 countries in the World Health Organization (WHO) European Region on six selected problem statements.

The survey asked countries which areas (presented as statements) related to child and adolescent health they considered a problem in their national context. **Figure 1** presents the top three statements identified as problems by most countries (right side) and statements countries did not identify as problems (left side) although evidence suggests these to be problematic in many countries [1,2,5,7-12].

Over 80% of the surveyed countries considered adolescent mental health and overweight and obesity as problem areas, and 69% of the countries considered low breastfeeding rates as being a national problem. Support for parenting skills and unnecessary hospitalizations remained low priorities: less than 40% of countries agreed that they are problem areas. Only 18% of the countries saw out-of-pocket payments for health care as a problem (see list of problem statements and results in Appendix S3 in the **Online Supplementary Document**).

### Governance and strategy

**Figure 2:**
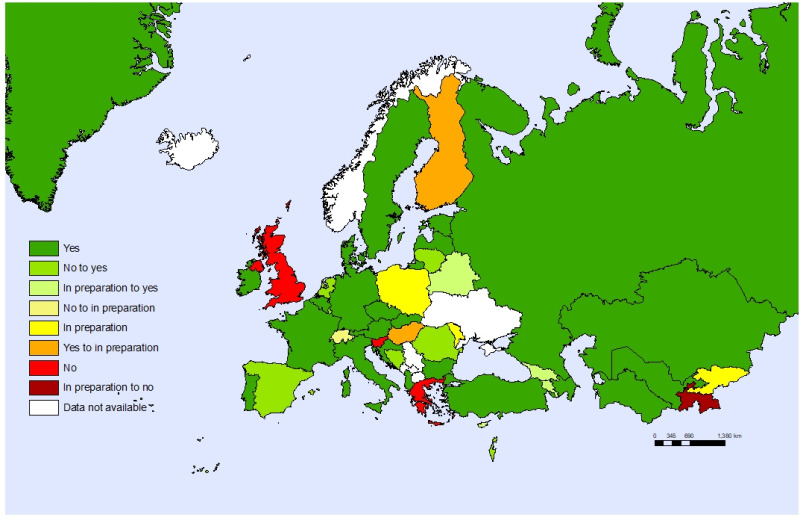
Countries having or preparing a child and adolescent health strategy in 2017 vs. 2020.

Forty-one out of 45 Member States reported to have adopted a child and adolescent health strategy or having one in preparation. **Figure 2** shows the changes observed between 2017 and the 2020 surveys. Twelve countries that did not have or were in the stage of preparation for a strategy in 2017 had a strategy in place in 2020. In most cases, the national child and adolescent health strategy was part of a wider strategy that covered either other age groups or other sectors.

**Figure 3:**
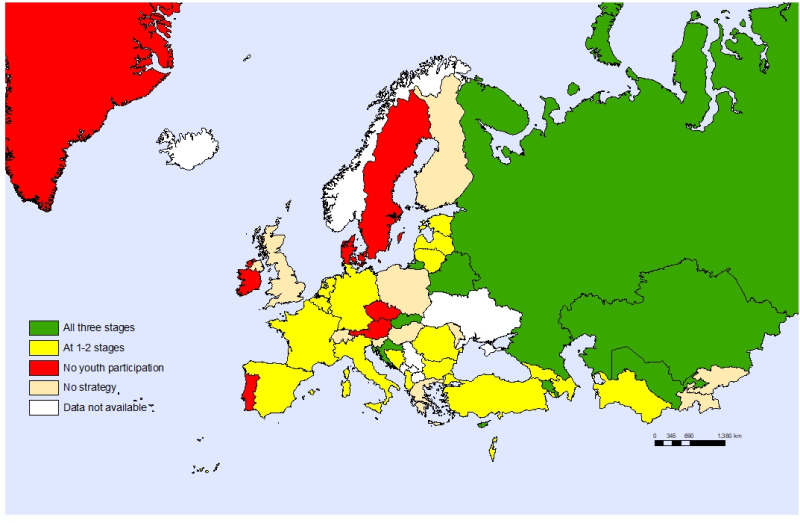
Countries that involved young people in the review, development, and implementation stages of their child and adolescent health strategy, 2020, by individual countries.

Only eight countries (18%) involved children and adolescents in the review, development and implementation stages of their child and adolescent health strategies. Twenty countries (44%) included them in one or two of these stages and six countries (13%) did not involve children at all, with 11 countries not reporting what they did in this regard (**Figure 3**).

### Reporting on child and adolescent health

Almost two-thirds of countries (62%) publish results related to child and adolescent health as part of their reporting for the SDGs and 35 countries (78%) report that health has been a consistent part of their reporting under the United Nations Convention on the Rights of the Child (UNCRC) [4].

Most countries disaggregate health service utilization data of children by geographic location and sex (35 and 32 countries, respectively) and almost half (22 countries) by socioeconomic background. Breaking down the data by migrant status and ethnicity is less common, with only nine countries providing statistics on the migrant status and only seven on ethnic background. Sixteen countries (35%) report that they systematically collect information on the health of migrant and refugee children. In 2017, only nine countries (20%) provided statistics on the number of unaccompanied, underage migrant children in their country. Countries in the region rarely collect data on children in institutional care with only 12 out of 45 countries providing data on the rate of children and adolescents in institutional care.

### Provision of care and health systems for children

Countries across the European region have different systems in place to provide primary health care (PHC) to children and adolescents: general practitioner (GP)-led, paediatrician-led, or a mixed system where different health care professionals provide care. PHC providers of choice differed by age of the patient.

Paediatricians are reported to provide well-child visits in 15 countries (33%) and care for sick children in 17 countries (38%). For adolescents, GPs are the main primary care providers for routine check-ups/preventative care visits in 17 countries (38%) and for sick adolescents in 19 countries (42%). For children with complex care needs, 25 countries (58%) reported offering care through a mix of specialist physicians including community and school nurses.

**Figure 4:**
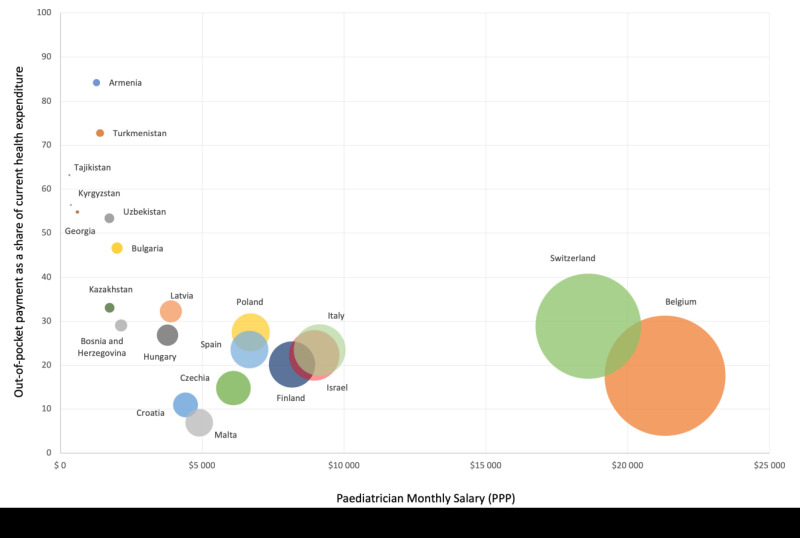
Average monthly salary of paediatricians (adjusted for purchasing power parity (PPP)) and out-of-pocket expenditure as a percentage of current health expenditure, by country. Note: Bubble size refers to paediatricians’ monthly salaries in international dollars (PPP).

Salaries of GPs, paediatricians and nurses, reported by countries, vary substantially across the region, even when adjusting for purchasing power parity (PPP) (**Figure 4**). Tajikistan reported the lowest salary for paediatricians, Switzerland and Belgium indicated 6-7 times higher salaries, even when adjusting for PPP. Countries where paediatricians had lower salaries had higher proportions of out-of-pocket payments. Most countries (89%) did not collect data on out-of-pocket and informal payments for health care and medicines specifically for children and adolescents. Despite the absence of data, 82% of countries did not consider out-of-pocket payments a problem. In 25 countries, dental care for children was fully covered under national health insurance, while 14 countries reported partial coverage only. The remaining countries have no coverage.

**Figure 5:**
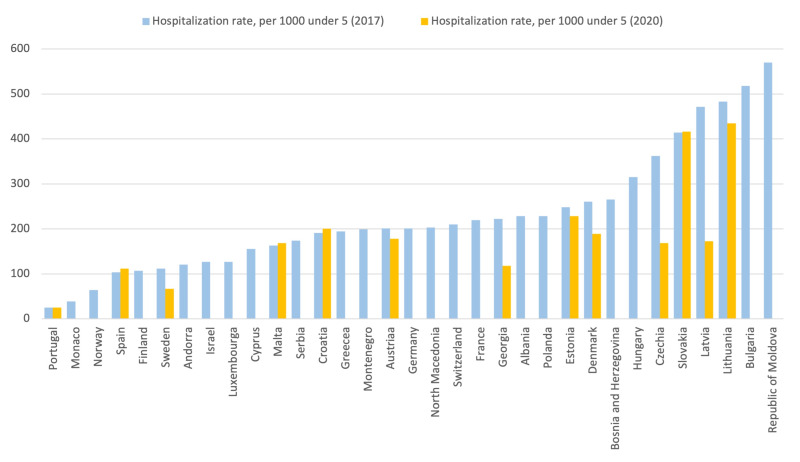
Hospitalization rates under 5 years, 2020 and 2017, by individual countries.

Hospitalization rates for children under five varied considerably across countries (**Figure 5**). In the problem statements, most countries (67%) did not consider unnecessary hospitalization for children or adolescents as a national problem.

Countries reported that the age cut-offs for allowing access to medicine without co-payment vary, ranging from 1 to 18 years or beyond in case of a young adult enrolled in secondary or university education. Most (39 countries) have a list of essential medicines that are reimbursable. Thirty-three countries included antibiotics in paediatric formulation. While antibiotics were widely available, other drugs to manage emergencies in children, such as seizures or severe pain were not always readily available at the PHC level. For the treatment of emergencies in children, just over half of the countries (58%) have diazepam or other rapid-acting benzodiazepines available in PHC facilities, and one-third of the countries (36%) have morphine or other opioids.

### Supporting early childhood development

Only 39% of countries in the European Region considered delayed identification of children with developmental difficulties as a problem. Access to and availability of antenatal and newborn screening programmes, important for the early identification and management of risks for development, varied across the region. Thirty-five countries provided screening for phenylketonuria, 34 for congenital hypothyroidism and 27 for hearing based on objective methods for all infants. Almost all (93%) countries reported routine assessment for developmental difficulties in children, but less than 25% collected and reported on the SDG indicator 4.2.1 (proportion of children under five years of age who are developmentally on track in health, learning and psychosocial well-being).

### Nutrition

**Figure 6:**
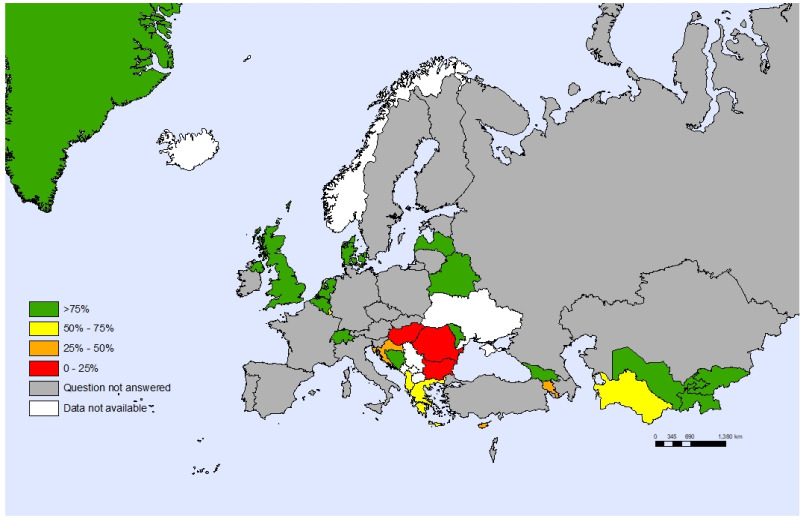
The prevalence of breastfeeding initiation within the first hour of birth.

The region has the lowest exclusive breastfeeding rates (25%) and proportion of countries in the world with comprehensive legislation according to the “International Code of Marketing of Breast-milk Substitutes” [10]. This is in line with the survey finding that 69% of responding countries consider low breastfeeding rates a national problem area. Initiation of breastfeeding within one hour of birth did not feature prominently within the region, with only 24 countries (53%) reporting doing it. The rates varied widely, lowest in Bulgaria (10%) and highest in Denmark (99.5%) (**Figure 6**). Two-thirds (32 countries) of countries did not have regulations for marketing of complementary feeding products for children 6-24 months and did not collect data on this. One-third (33%) of countries in the region did not have legislation against marketing of unhealthy foods and beverages to children. One-third (33%) of the responding countries also did not have legislation in place that targets the availability of unhealthy foods in schools, while over one in four countries allowed the sale of unhealthy foods via vending machines and school kiosks.

### Rights and participation of children and adolescents

**Figure 7:**
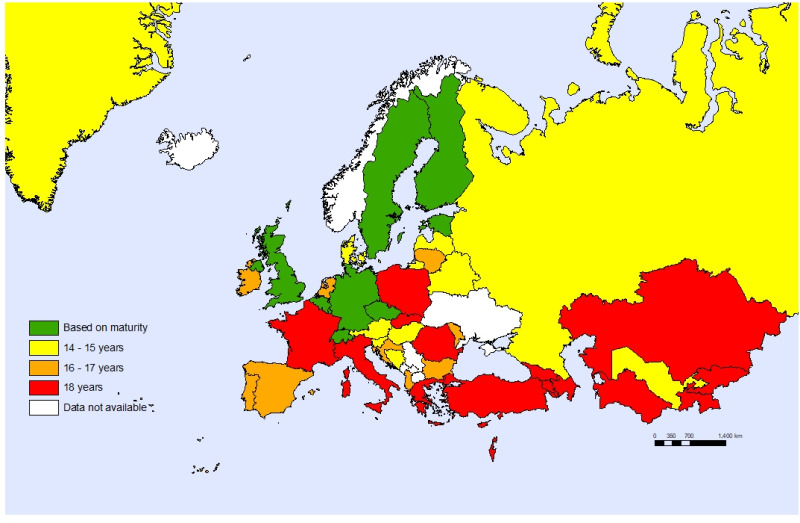
Age at which adolescents can access health care without parental consent.

**Figure 8:**
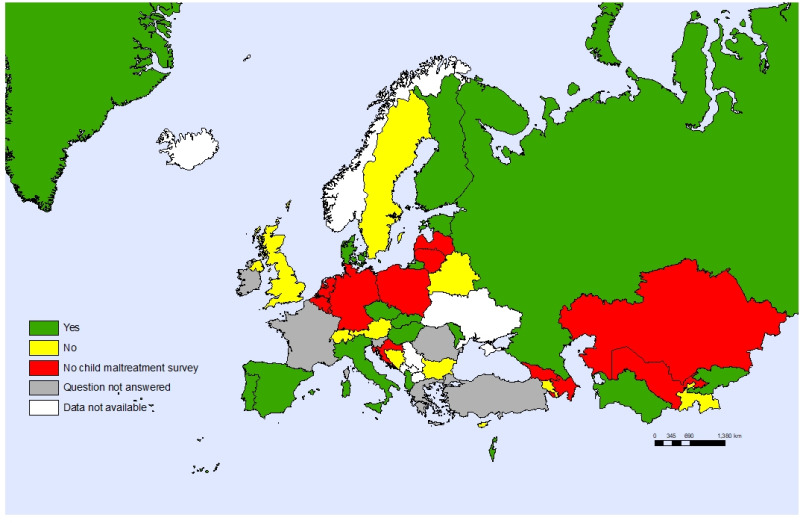
Countries having population-based surveys on child maltreatment completed also by children.

Sixteen countries (36%) reported that only adolescents aged 18 years old can access health care without parental consent and only ten countries (22%) report that adolescents are allowed to consent to health care without parental consent, based on an assessment of their maturity (**Figure 7**). Only one-third (33%) of the countries conduct surveys on violence against children that include a part completed by children themselves (**Figure 8**).

### Mental health and health promoting environments

**Figure 9:**
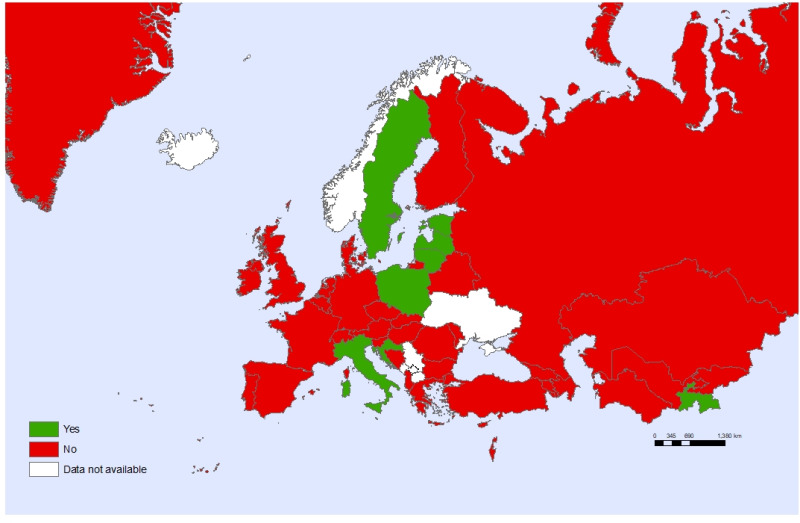
Countries able to provide estimates on the number of prescriptions issued for attention deficit hyperactivity disorder (ADHD), autism and depression.

Most countries (89%) considered adolescent mental health to be a key challenge in their national context. There was a substantial lack of country-level data on mental health indicators, such as the proportion of adolescents treated for common mental health conditions or prescription rates of medications used to treat attention deficit hyperactivity disorder (ADHD), autism and depression (**Figure 9**). While only seven countries were able to provide prescription rates in 2017, 11 countries provided numbers in 2020. Over two-thirds (71%) of countries offered community-based early intervention programmes for young people experiencing a first episode of a severe mental health problem such as a psychosis. Two-thirds (67%) of countries have a strategy for health-promoting schools, 71% have a school curriculum for health education, and 24 countries have a policy on employing nurses in the school environment.

### Sexual health

**Figure 10:**
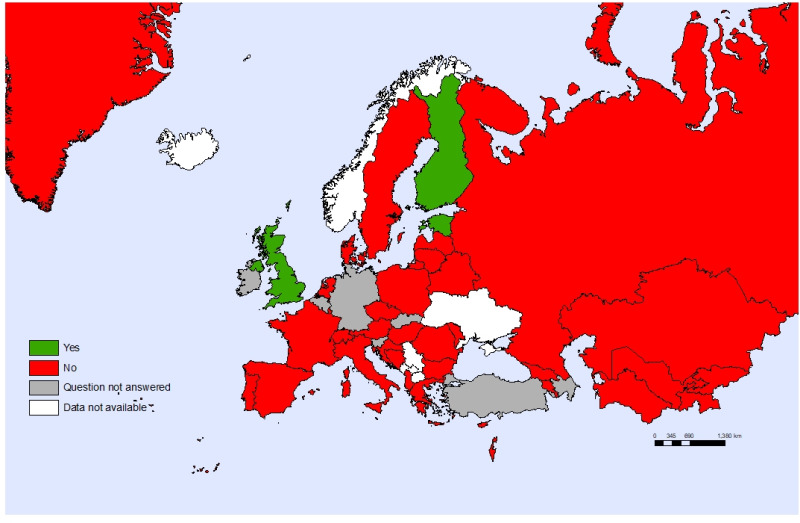
School nurse dispensing contraceptives without a doctor’s prescription, by country.

Most (82%) countries have a policy on sex education in schools, but only 26 countries collected information about adolescents’ knowledge of sex. Thirty-two countries reported that adolescents under 18 years have access to contraceptives without parental consent, but only 23 countries provided free emergency contraception for adolescents. School nurses are not allowed to dispense contraceptives (in 34 countries, **Figure 10**) or emergency contraception (32 countries) without a doctor’s prescription. Most countries (71%) reported providing free diagnostic tests for sexually transmitted infections (STIs) to adolescents, but only 14 countries legally ensured confidentiality while accessing treatment for STIs.

## DISCUSSION

Reaching the end year of the European strategy 2015-2020, 45 countries in the European region reported their progress on child and adolescent health towards meeting the 2030 Agenda. This paper observed both improvements and areas where more improvement is needed with regards to child and adolescent health in Europe.

The series of problem statements (Appendix S3 in the **Online Supplementary Document**) gave a sense of which topics countries saw as a priority and those that were not yet on their radar when it came to child and adolescent health. One way that countries can address the problem areas is through the adoption of comprehensive health strategies for this age group as national strategies, involving all stakeholders in their articulation. This will lead to a planned agenda to execute coordinated actions for improvement.

The trend in the region shows that many countries have or are planning for national child and adolescent health strategies, but some countries that were planning strategies in the last survey in 2017 have dropped out of the effort, which is concerning. Very few countries involve children and adolescents in developing such national strategies, although children and adolescents should have a say, be heard, and be key stakeholders of national strategies that address their health.

Collecting reliable data and disaggregating data by age, sex, social or ethnic background for policy decisions is a critical government role in order to address existing gaps and meet needs. Reporting collected data on indicators related to the SDGs or the UNCRC can be a source of accountability for governments in the adoption of global commitments on health and rights. Yet, the lack of data collection and reporting remain a key concern for the region. Data alone does not affect change. The survey findings point to a need for greater coordination across policy/intervention areas covered in national strategies. To align with global commitments, countries need to adopt a culture of evidence, strengthen their health information systems and embrace digitalization, make available required resources both financial and human, invest in health services and pre-service and post-graduate education, enforce evidence-based rules and regulation.

Early childhood development lays the foundations for health, development and well-being later in life. Infants and young children depend on their caregivers and health services to recognize, assess and respond to their needs for health, nutrition, safety, early stimulation and learning. More than five million children were at risk of not reaching their full developmental potential in the region before the events of 2020-2022 [13]. A European framework aims to inform countries on measures they can take to enable young children to reach their full potential equally (see Appendix S4 in the **Online Supplementary Document**) [12]. Adequate infant and young child feeding practices are crucial for the prevention of under- and overweight, obesity and diet-related non-communicable diseases. The findings of the survey are alarming in light of the growing number of children with obesity in the Region, which tripled in many European countries since the 1980s [8,14-16]. Efforts should be made to implement policies across the life cycle, from promotion of breastfeeding to healthy behaviours in schools, such as eating behaviours and physical activity.

Evidence emerging from applied health systems assessments in the region suggests low trust in PHC providers in many settings and the preference for second-level specialized care [11]. Seeking hospital care for something that could be attended by a PHC provider should be avoided both due to potential harm for the child and costs for families and health systems. While there is no evidence whether any particular PHC system (e.g. GP-led, paediatrician-led, or a mixed system) is superior to another, countries must ensure that all health providers seeing children and adolescents are professionally competent and use child- and family-centred practices. The WHO European Region has recently launched the Pocket Book of Primary Health Care for Children and Adolescents to guide health care providers to deliver on the promise of quality primary health care (see Appendix S5 in the **Online Supplementary Document**) [17]. Relatedly, hospitalization rates across the region varied considerably. High rates may indicate over-hospitalization of children who could safely be managed as outpatients, whereas very low rates may indicate a problem with access to hospital care. While the causes of the variability were beyond the scope of the survey, high rates could be at least partially explained by financial incentives for admitting patients to hospitals contributing to unnecessary hospitalization in countries [11].

Out-of-pocket and informal payments for health care and medicines for children and adolescents may be more frequent than currently recognized [11]. National policies commonly state that children are exempted from co-payments for medicines and services, but the age definition of “children” varies widely leading to extra expenses for many children. Low salaries may partially be contributing to the prevalence of out-of-pocket and informal payments as well (**Figure 4**) [11]. Quality of care may be adversely impacted when financial considerations of doctors play a role, rather than adhering to evidence-based practice and the best interest of the child. Revising the health financing systems where they are incentivizing unnecessary hospitalizations or treatments is vital to move towards a cost-effective health system.

Countries have an obligation to children under the UNCRC, meaning that “the child, by reason of his physical and mental immaturity, needs special safeguards and care, including appropriate legal protection, before as well as after birth” [18]. At the same time the UNCRC gives children and adolescents the right to express their opinion about their health and be active participants to address issues affecting them. It requires that an assessment of maturity, on a case-by-case basis, should permit them giving consent to medical consultations and treatments, without involving their parents [18]. In many countries, children under 18, even when they demonstrate maturity, are not allowed to seek care without parental consent and often do not have access to sexual and reproductive health services.

Violence and injuries are a major public health and human rights concern and are major drivers of mortality and morbidity in children and adolescents [10]. There is a significant burden of morbidity and mortality attributable to mental ill-health and poor mental well-being [9]. It is encouraging that the majority of countries recognizes that adolescent mental health as a problem area. It is critical for countries to put in place structures and policies that support children and adolescent’s mental health and well-being. Schools have an important role to play for promoting and maintaining the well-being of children and serve as a platform to increase access to health services.

Sexual health is fundamental to overall health and well-being, with adolescence being a critical period in which the first intercourse usually occurs. However, adolescents’ sexual health is considered a sensitive issue in some countries of the WHO European Region. Thus, adolescents face barriers in accessing sexual reproductive health services. One way to remove access barriers for adolescents is to allow school nurses to provide health counselling and dispense contraceptives, when required and indicated. Most importantly, countries must ensure confidentiality and accessibility for adolescents who seek these health services.

Age, sex, socioeconomic status, and culture remain strong determinants in access to care for children and adolescents and those in lower-income households experience more barriers in access to health care [11]. More focus is needed on migrant and refugee children’s access to health care especially now when the crisis in Ukraine has forced many to seek refuge in neighbouring and other countries. Refugee children and adolescents come from diverse backgrounds and their exposure to multiple risk factors before departing from their country of origin, during the journey, and in the country of destination can impact their well-being and development into adulthood. When it comes to institutional care for children and adolescents, they should only be separated from their families and communities and placed in institutional care when care cannot be provided otherwise and when absolutely necessary.

### Strengths and limitations

The paper provides a basis for further discussion in countries on how to improve the situation for children and adolescents. To overcome the potential unreliability of self-reported data by ministries of health, we triangulated the responses obtained from the ministries with other publicly available data and surveys, such as the applied health system assessments [11], the Childhood Obesity Survey Initiative (COSI) [8], and the Health Behaviour in School-aged Children (HBSC) survey [19] (see Appendix 6 in the **Online Supplementary Document**) and cross-referenced the data. Complementary materials such as the more comprehensive report on the results [6] and the database of the survey available through WHO European Health Information Gateway website [20] can be accessed by countries to guide their response to identified problems faced by children and adolescents in the region.

### Moving forward

Childhood and adolescence represent particularly sensitive developmental periods with unique challenges that influence lifetime health, well-being, and prosperity, both at the individual and societal level. Investment in these age groups presents an opportunity to address long-term inequalities and achieve more equitable social and economic benefits for the region’s populations. Any future actions to improve the lives of children and adolescents should be embedded in WHO’s and countries commitment to human rights and should be structured around promoting healthier populations, enabling universal access to services and protecting children in emergencies. The COVID-19 pandemic has painfully shown and exacerbated pre-existing weaknesses in most health care systems, often leaving children and adolescents behind. The war in Ukraine adds an immense burden on the children affected, and on others indirectly. Along with the redesign of health systems with adequate financing, a joint effort among ministries of health, other ministries, and stakeholders in the region to implement actions to improve the health of its children and adolescents is critical to ensure that the progress made over the past decades are not wiped out completely by the recent events. To achieve universal health coverage (UHC) for children, designing health systems around and for children will be required and evidence-based quality health services for children and adolescents must be delivered.

## CONCLUSIONS

This paper illustrates progress made in child and adolescent health in the WHO European Region until 2020 and the areas where additional work is required to reach the SDGs. Countries can review and use collected data to compare their achievements and address gaps. The survey was undertaken before the COVID-19 pandemic and the war in Ukraine, which will likely have exacerbated many of the observed problems and potentially reverse many of the gains reported. The results have to be used for the development of a new European roadmap for child and adolescent health to ensure that the threats posed by the COVID-19 pandemic and the war in Ukraine and their side effects are mitigated and children and adolescents in the region are allowed to achieve their full potential.

## Additional material


Online Supplementary Document
