# Supplementary material for: Child and adolescent health in Europe: Towards meeting the 2030 agenda
Source: J Glob Health. 2023 Jan 20;13:04011. doi: 10.7189/jogh.13.04011 (PMC9850873; doi:10.7189/jogh.13.04011)
Supplement: Online Supplementary Document [file jogh-13-04011-s001.pdf]

ONLINE SUPPLEMENTARY DOCUMENT

Title: Child and adolescent health in Europe: towards meeting the 2030 agenda

Authors: Minhye Park, Sanja Budisavljević, Aixa Y. Alemán-Díaz, Susanne Carai, Katharina Schwarz, Aigul Kuttumuratova, Lei B. Jobe, Vivien Hülsen, Yae Eun Lee, Eileen Scott, Ross Whitehead, Martin W. Weber

Appendix S1. Moving forward on child and adolescent health in the European region

The European Programme of Work (EPW) "United Action for Better Health" (2020–2025) sets three core priorities: 1) moving towards universal health coverage (UHC); 2) protecting people better against health emergencies; and 3) promoting health and well-being.

Based on the EPW's core priorities, a new roadmap for European child and adolescent health was devised (Figure 1). This effort builds on the 2015-2020 European strategy for children and adolescents in order to ensure healthy lives and well-being for all children and adolescents and to meet the SDGs in the WHO European Region by 2030. The new roadmap rests on the guiding principles of a life-course approach to health: policy and planning that is based on an evidence-informed approach; accountability; and a framework that respects the rights of children and adolescents, especially their right to health. This roadmap provides countries with an approach to addressing region-specific causes of mortality and morbidity.

By 2030, it is envisioned that all children and adolescents in the European Region would be aware of their rights to physical and mental health and well-being, and will be able to fully participate in shaping prosperous and sustainable societies.

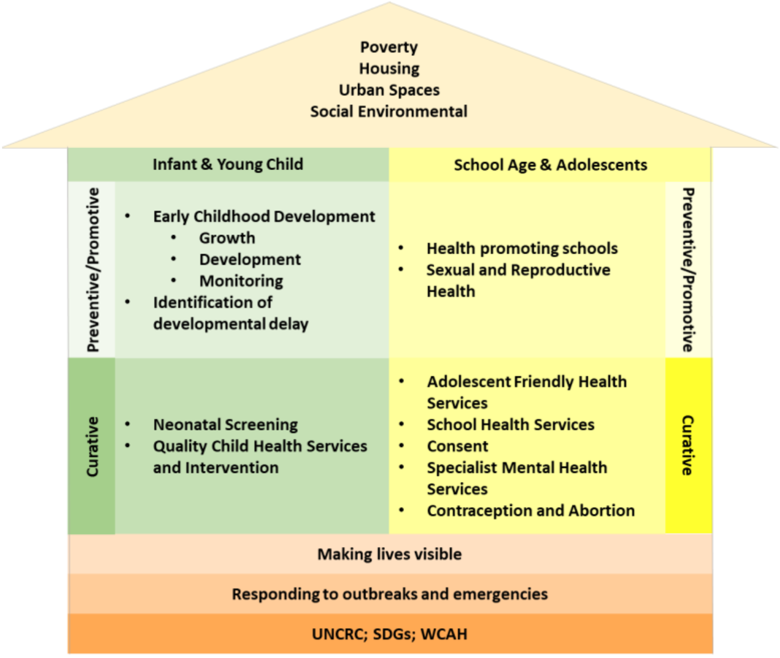

Figure S1. New roadmap for children and adolescents in the European region

Appendix S2. Survey questionnaire

European Child and Adolescent Health Strategy – 2020 Survey

Please note that this document is only for guidance. Please fill in the electronic version.

The purpose of this questionnaire is to collect information on the implementation of the European Child and Adolescent Health Strategy 2015–2020. The content is based on the strategy and associated tools. Information on child and adolescent health issues in countries was obtained by a previous survey in 2017 that was submitted to the regional committee in 2018. In 2020 the Secretariat will present a final report at its 70th session which requires us to collect additional data.

Completing the survey

Begin the survey by clicking **Next**. The survey can be paused and returned to at any time, your responses will be saved every time you click **Next**. Previously saved answers and comments can be altered by clicking **Previous** until you click **Submit** on the last page.

You can download the full version of the survey [here]. Further information about how to complete the survey can be found in the information guide [here]. The survey is available in English and Russian. The language can be changed using the menu in the top right corner.

You will not be able to use Internet Explorer (IE) to complete this survey. Please use one of the following browsers: Chrome, Firefox or Opera.

Submission of the survey

Please submit the survey before **Monday, 6 January 2020** so that they can be incorporated in the analysis.

Helpdesk

If you require any support while completing the survey or have questions on how to complete it, please contact us at CAHsurvey2019@who.int.

1. Do you have a national strategy for child and adolescent health and development that has been adopted within the last five (5) years?

Please review your answer (confirm/change) as appropriate.

☐ Yes ☐ In preparation ☐ No

Please explain the age range the strategy covers and provide a link to the relevant document(s).

The age range explanation

Link

1.a Is it a "stand-alone" strategy or is it part of another strategy? *For example, a general children's strategy; an overall population health strategy.*

Please review your answer (confirm/change) as appropriate.

☐ Stand-alone. ☐ Part of another strategy

Please explain.

1.b Please indicate at which stage youth have been involved in the child and adolescent health strategy? (check all that apply)

Involvement could include participating in hearings, policy planning, design or running of youth-friendly services, etc.

Please review your answer (confirm/change) as appropriate.

☐ Review ☐ Development ☐ Implementation

Please explain.

1.c Have you recently reviewed the existing child and adolescent health strategy?

☐ Yes ☐ No

1.d Are there plans to review the existing child and adolescent health strategy?

☐ Yes ☐ No

2. Do you have a national strategy for sexual, reproductive, maternal, newborn health that has been adopted within the last five (5) years?

☐ Yes ☐ No

Please provide a link.

3. Do you publish Sustainable Development Goals (SDG) indicators related to child and adolescent health in your country?

☐ Yes ☐ No

Please provide the link or comment:

4. When developing policies in the following areas, did your country consider them in terms of their impact upon the health of children and adolescents? (check all that apply)

Please review your answer (confirm/change) as appropriate.

☐ Justice

☐ Education

☐ Physical health

☐ Economic/financial

☐ Family/maternity

☐ Nutrition

☐ Sexual and reproductive health

☐ Other

Please describe.

5. The next child and adolescent health strategy is meant to address priorities around child and adolescent health. The following problem statements stem from our monitoring work of the current strategy.

Which of the following statement(s) are of priority in your country? (check all that apply)

☐ Adolescent access to health services

☐ Over prescription of antibiotics

☐ Unnecessary admission to hospitals

☐ Substantial out-of-pocket payments for health care of children and adolescents

☐ Adolescent mental health

☐ Low exclusive breastfeeding rates

☐ Aggressive promotion of unhealthy foods for children

☐ Increasing number of children who are overweight or obese

☐ Schools are not a health promoting environment

☐ Children are not taught in schools what they need to know about their health, present and future

☐ Parents are not taught parenting skills

☐ Late identification of children with developmental difficulties

☐ Low or decreasing vaccination coverage

☐ High rates of road and other injuries

☐ Left-behind children whose parents working abroad

☐ Increasing gambling and gaming among adolescents

6. Is health service utilization analysed in terms of the following differences? (check all that apply)

☐ Rural, suburban and/or specific urban areas

☐ Socioeconomic background

☐ Ethnic background

☐ Migrant status

☐ Sex

7. To whom would children under 5 generally present when sick (according to your system)?

☐ General Practitioner ☐ Paediatrician ☐ Other

7.a What proportion of these children would be seen by this provider? (%)

7.b Is this data based on estimates or statistics?

☐ Estimates ☐ Statistics

8. To whom would adolescents generally present when sick (according to your system)?

☐ General Practitioner ☐ Paediatrician ☐ Other

8.a What proportion of these adolescents would be seen by this provider? (%)

8.b Is this data based on estimates or statistics?

☐ Estimates ☐ Statistics

9. To whom would children under 5 generally present for well-child visits? (e.g. routine check-ups, developmental monitoring, preventative care)

☐ General Practitioner ☐ Paediatrician ☐ Other

9.a What proportion of these children would be seen by this provider? (%)

9.b Is this data based on estimates or statistics?

☐ Estimates ☐ Statistics

10. To whom would adolescents generally present for routine check-ups or preventative care visits?

☐ General Practitioner ☐ Paediatrician ☐ Other

10.a What proportion of these adolescents would be seen by this provider? (%)

10.b Is this data based on estimates or statistics?

☐ Estimates ☐ Statistics

11. Who is the primary care provider for children with complex care needs? (e.g. cerebral palsy, chronic conditions)

☐ General Practitioner ☐ Paediatrician ☐ Other

12. Do you have a system to coordinate plans and share key information about the care needs of children and adolescents with complex needs across multiple sectors? (e.g. children with cerebral palsy, or autism, in terms of access to school for children with disabilities, or access to social services)

☐ Yes ☐ No

Please describe this system and how these children are transitioned into adult care.

13. Do you have any guidelines for paediatric palliative care in your country?

☐ Yes ☐ No

Please describe.

comment

14. What is the age of consent for access to healthcare for adolescents without parental consent?

The prefilled information was compiled from different data sources for your country. Please confirm/revise.

Age of consent or based on maturity:

Comment.

15. If adolescents wish to have their access to care remain confidential, how does your country ensure that parents are not made aware of the services their adolescent children receive? (e.g. sexual reproductive health or mental health services, i.e. by receiving an invoice)

Please describe.

16. Do you collect data on out-of-pocket health expenses for children and adolescents?

☐ Yes ☐ No

16.a What is the average percentage of out-of-pocket health expenses for children under 5 and adolescents? Please also provide a link.

	**Average Percentage**	**Link**
Children under 5		

Adolescents	

17. Do you collect information on informal payments to health providers?

☐ Yes ☐ No

Please describe how you collect this information.

18. Is dental care for children and adolescents covered under the national health insurance programme?

☐ Yes ☐ Partially covered ☐ No

Please describe what is covered and until what age.

19. Do you undertake routine assessment of developmental difficulties in children?

☐ Yes ☐ No

Please describe at what age(s).

20. Do you undertake routine health assessments of school-aged children?

☐ Yes ☐ No

Please describe at what age(s).

20.a What do these routine assessments include?

21. Do you perform the following screenings on newborns and young infants?

The list below was compiled through literature review.

Test	Screening performed on		Explain which subgroup	Estimated coverage of screening (%)
	All children	Subgroup of children		
Hearing with objective methods	☐	☐		
Metabolic diseases (list)				
CH – Congenital hypothyroidism	☐	☐		
PKU – Phenylketonuria	☐	☐		
MCADD – Acyl-CoA delydrogenase deficiency	☐	☐		
GA1 – Glutaric acidemia type 1	☐	☐		
CF – Cystic fibrosis	☐	☐		
CAH – Congenital adrenal hyperplasia	☐	☐		
NSUD – Maple syrup urine disease	☐	☐		
IVA – Isovaleric acidemia	☐	☐		
LCHADD – Long-chain 3 hydroxyacyl CoA dehydrogenase deficiency	☐	☐		
VLCADD – Very long-chain 3 hydroxyacyl CoA dehydrogenase deficiency	☐	☐		
Others	☐	☐		

22. What is the average monthly salary before tax (in local currency) of:

	Salary (in local currency)
General paediatricians	
General practitioners/Family doctors	
Nurses	

23. Does your country have a system of home visiting nurses (e.g. nurse practitioners, patronage nurses, community nurses, district nurses) to support child and adolescent health?

☐ Yes ☐ No

Please describe the services they provide.

23.a Do these nurses/midwives have authority to initiate treatment or prescribe medicines?

☐ Yes ☐ No

Please describe the services they provide.

23.b Does your country have a system for home visits of newborns and young infants?

☐ Yes ☐ No

Please describe the services they provide.

24. Is adolescent health included in pre-service training of the following: (check all that apply)

☐ Doctors ☐ Nurses

25. What is the total hospitalisation rate for children under 18?

Please update your answer, indicate either number or rate.

	**Number**	**Rate (per 1000 under 18)**	**Year of latest data**
Under 5			
Age 5-9			
Age 10-14			
Age 15-18			

26. What is the hospitalisation rate for pneumonia for children under 5?

Please update your answer, indicate either number or rate.

Number

Rate (per 1000 under 5)

Year of latest data

27. Is treatment for pneumonia cheaper for the patient if they are admitted to the hospital as compared to outpatient treatment?

☐ Yes ☐ No

Comment.

28. Are hospital payment systems linked to a minimum stay for children in hospitals?

☐ Yes ☐ No

Comment.

29. Do you collect systematic information on maternal alcohol consumption?

Please review your answer (confirm/change) as appropriate.

☐ Yes ☐ No

Please describe and include link(s) to relevant documentation.

Description

Link(s)

30. Please provide breastfeeding rates for:

	**Rate (%)**	**Year of latest data**

Exclusive breastfeeding up to 6 months	
Breastfeeding initiation within the first hour	

31. Is there a national policy for the provision of human donor milk for babies whose mothers cannot produce sufficient breastmilk?

Please review your answer (confirm/change) as appropriate.

☐ Yes ☐ No

32. Is there an effective mechanism for inter-sectoral and multi-stakeholder planning and monitoring of child and adolescent mental health services (e.g. including family/carer groups and other civil society organizations)?

☐ Yes ☐ No

Please describe and include how often it meets per year.

33. In the last calendar year, what is the rate of children and adolescents under 18 treated by a mental health professional for: Attention deficit hyperactivity disorder, autism and depression?

Rate per 1000 children or adolescents

	**Rate**	**Year of latest data**
Attention deficit hyperactivity disorder		
Autism		
Depression		

34. Do you offer community services for early intervention and continuing support to young people with a first episode of a severe mental health problem? (e.g. psychosis)

☐ Yes ☐ No

Please describe what the services include.

35. Do you collect data on the marketing of complementary feeding products for children 6-24 months?

Please review your answer (confirm/change) as appropriate.

☐ Yes ☐ No

36. Do you regulate marketing of complementary feeding products for children 6-24 months?

☐ Yes ☐ No

37. Do you collect data on the prevalence of overweight/obesity of children under 5?

Please review your answer (confirm/change) as appropriate.

☐ Yes ☐ No

37.a Please provide the prevalence of overweight, obesity and underweight/wasting of children under 5 in your country?

	**Prevalence**	**Year of latest data**
Overweight		
Obesity		
Underweight/wasting		

38. Do you have any laws or regulations which restrict marketing to children?

Please review your answer (confirm/change) as appropriate.

☐ Yes ☐ Planned ☐ No

Please describe what type of marketing is/will be restricted.

38.a Does it include direct marketing via mobile phone or game platforms?

☐ Yes ☐ No

39. Do you have legislation that restricts the availability of unhealthy foods in schools?

Unhealthy foods include foods with high sugar, salt or fat content, particularly those high in saturated and trans fats.

Please review your answer (confirm/change) as appropriate.

☐ Yes ☐ No

Please describe the legislation and include link(s) to relevant documentation.

Description

Link(s)

39.a Do you allow the sale of unhealthy foods in schools, such as soft drinks, chips/crisps, sweets, via vending machines or kiosks?

☐ Yes ☐ No

40. Are children diagnosed or in contact with tuberculosis (TB) routinely hospitalized or placed in sanatoria?

☐ Hospitalized ☐ Sanatoria ☐ No such mechanism

Please describe when children are hospitalized/placed in sanatoria.

41. How many children under 3 and under 18 are in long-term institutional care (e.g. due to disability or under the protection of child welfare services)? Includes social services as well as health service institutions but excludes short-term hospitalisations.

	**Number**	**Rate (per 1000 children)**
Under 3		
Under 18		

42. What proportion of children under 3 in institutional care are there for the following reasons? Responses should add up to 100 percent within each age category.

Intellectual and developmental disabilities

Court order (child welfare/protection)

Other

42.a What proportion of children under 18 in institutional care are there for the following reasons? Responses should add up to 100 percent within each age category.

Intellectual and developmental disabilities

Court order (child welfare/protection)

Court order (juvenile or youth offenders)

Other

43. What proportion of children and adolescents in institutional care have care plans to place them in the community (e.g. with foster families, sheltered housing)?

☐ Less than 10%. ☐ 10-20%. ☐ 20-50% ☐ 50-80% ☐ More than 80%

44. Do children in your country complete child maltreatment population-based surveys?

☐ Yes ☐ No ☐ No child maltreatment population-based survey

45. Do you collect systematic information on the health of migrant and refugee children?

Please review your answer (confirm/change) as appropriate.

☐ Yes ☐ No

Please describe and include link(s) to relevant documentation.

Description

Link(s)

46. Please provide statistics on the number of unaccompanied, underage migrant or refugee children in your country.

	Number
Migrant	
Refugee	

47. Do you have data on the number of children temporarily living under the care of extended family when parents are out of the country for extended periods (e.g. for work, studies)?

☐ Yes ☐ No

Please provide number or rate (per 1000 children):

48. Do you have a policy to have nurses in schools?

☐ Yes ☐ No

49. Do you have a curriculum for health education in schools?

☐ Yes ☐ No

Please provide a link.

50. Do you have a strategy on health promoting schools?

☐ Yes ☐ No

51. Do you have a reimbursable medicines list?

☐ Yes ☐ No

51.a Does it include antibiotics in paediatric formulation?

☐ Yes ☐ No

52. Up to what age are medicines covered free of charge?

Age:

53. Please provide the number of prescriptions for mental and behavioural disorders for children and adolescents under 18 for:

	Number of prescriptions	Year of latest data
Attention deficit hyperactivity disorder		
Autism		
Depression		

54. Are the following drugs available in primary health care facility for the treatment of emergencies in children?

	Yes	No
Morphine (or other opioids)	☐	☐

Diazepam (or other rapid acting benzodiazepines)	☐	☐

Comment.

55. Do you have a policy of having sexuality* education in schools?

☐ Yes ☐ No

Please describe.

*The concept of sexuality education in this survey does not refer to only aspects of sexual preference(sexuality) but also includes education on general sexual health.

56. Does sexuality education* in school include issues of homosexuality and gender diversity?

☐ Yes ☐ No

Please describe.

*The concept of sexuality education in this survey does not refer to only aspects of sexual preference(sexuality) but also includes education on general sexual health.

57. Do you collect information about children's and adolescent's knowledge on sexuality?

Please review your answer (confirm/change) as appropriate.

☐ Yes ☐ No

Please describe and include link(s) to relevant documentation.

Description

Link(s)

58. Are sexual and reproductive health services for adolescents <u>under 18</u> free of charge?

☐ Yes ☐ No

59. Can adolescents <u>under 18</u> receive contraception without parental consent?

☐ Yes ☐ No

59.a What is the age limit for receiving contraception without parental consent?

60. Can adolescents <u>under 18</u> receive <u>emergency</u> contraception without parental consent?

☐ Yes ☐ No

What is the age limit for receiving <u>emergency</u> contraception without parental consent?

61. Is emergency contraception available without prescription for adolescents?

☐ Yes ☐ No

62. Is emergency contraception free of charge for adolescents?

☐ Yes ☐ No

63. Can a school nurse dispense contraceptives without a doctor's prescription?

☐ Yes ☐ No

64. Can a school nurse dispense emergency contraceptives without a doctor's prescription?

☐ Yes ☐ No

65. Does your country undertake age and gender appropriate education that addresses sexual/intimate partner violence?

Please review your answer (confirm/change) as appropriate.

☐ Yes ☐ No

Please describe.

66. Can adolescents get diagnostic tests for Sexually Transmitted Infections (STI) free of charge?

☐ Yes ☐ No

66.a How do you ensure the confidentiality of adolescents accessing treatment for Sexually Transmitted Infections (STI)?

Appendix S3. Statements of problems affecting children and adolescents in Europe

Country	Adolescent access to health services	Over prescription of antibiotics	Unnecessary admission to hospitals	Substantial out-of-pocket payments for health care of children and adolescents	Adolescent mental health problems	Low exclusive breastfeeding rates	Aggressive promotion of unhealthy foods for children	Increasing number of children who are overweight or obese
Albania	Yes	Yes	No	No	Yes	No	Yes	Yes
Andorra	-	-	-	-	-	-	-	-
Armenia	Yes	Yes	Yes	No	Yes	Yes	Yes	Yes
Austria	No	No	No	No	No	No	Yes	Yes
Azerbaijan	Yes	Yes	No	No	Yes	Yes	No	No
Belarus	No	Yes	Yes	No	Yes	Yes	Yes	Yes
Belgium	No	Yes	Yes	No	Yes	Yes	No	Yes
Bosnia and Herzegovina	Yes	No	No	No	Yes	Yes	No	Yes
Bulgaria	Yes	No	No	No	Yes	Yes	No	Yes
Croatia	No	No	No	No	Yes	Yes	No	Yes
Cyprus	Yes	Yes	No	No	Yes	Yes	Yes	Yes
Czechia	No	Yes	No	No	Yes	Yes	Yes	Yes
Denmark	Yes	No	No	No	Yes	Yes	No	Yes
Estonia	Yes	Yes	Yes	Yes	Yes	Yes	Yes	Yes
Finland	Yes	No	No	No	Yes	No	No	Yes
France	Yes	No	No	Yes	Yes	Yes	Yes	Yes
Georgia	No	Yes	Yes	Yes	Yes	Yes	Yes	No
Germany	No	No	No	No	Yes	Yes	No	Yes
Greece	Yes	Yes	No	No	Yes	Yes	Yes	Yes
Hungary	Yes	Yes	Yes	Yes	Yes	Yes	Yes	Yes
Iceland	-	-	-	-	-	-	-	-
Ireland	No	No	No	No	No	Yes	Yes	Yes
Israel	Yes	Yes	Yes	No	Yes	Yes	Yes	Yes
Italy	Yes	Yes	Yes	Yes	Yes	Yes	Yes	Yes
Kazakhstan	Yes	Yes	No	No	Yes	No	Yes	Yes
Kyrgyzstan	Yes	Yes	Yes	No	Yes	No	No	Yes
Latvia	Yes	No	No	Yes	Yes	No	No	Yes
Lithuania	Yes	Yes	Yes	Yes	Yes	Yes	Yes	Yes
Luxembourg	No	Yes	No	No	Yes	Yes	No	Yes
Malta	No	No	No	No	Yes	Yes	Yes	Yes
Monaco	No	No	No	No	Yes	No	No	Yes
Montenegro	-	-	-	-	-	-	-	-
Netherlands	Yes	No	No	No	Yes	No	Yes	Yes
North Macedonia	-	-	-	-	-	-	-	-
Norway	-	-	-	-	-	-	-	-
Poland	No	No	No	No	Yes	Yes	Yes	Yes
Portugal	Yes	Yes	Yes	No	Yes	Yes	No	No
Republic of Moldova	Yes	Yes	Yes	No	Yes	Yes	Yes	Yes
Romania	Yes	Yes	Yes	Yes	Yes	Yes	Yes	Yes
Russian Federation	No	No	No	No	Yes	No	Yes	Yes
San Marino	-	-	-	-	-	-	-	-
Serbia	-	-	-	-	-	-	-	-
Slovakia	No	Yes	No	No	Yes	Yes	No	Yes
Slovenia	No	No	No	No	No	No	No	No
Spain	Yes	Yes	No	No	Yes	No	Yes	Yes
Sweden	No	No	No	No	No	No	No	No
Switzerland	Yes	Yes	No	No	Yes	Yes	Yes	Yes
Tajikistan	Yes	Yes	Yes	No	Yes	Yes	No	Yes
Türkiye	Yes	Yes	Yes	No	Yes	Yes	Yes	Yes
Turkmenistan	Yes	No	No	No	No	No	No	Yes
Ukraine	-	-	-	-	-	-	-	-
United Kingdom	No	No	No	No	No	Yes	Yes	Yes
Uzbekistan	No	Yes	No	No	No	No	Yes	Yes

		Adolescent access to health services (Yes)	(No)	Over prescription of antibiotics (Yes)	(No)	Unnecessary admission to hospitals (Yes)	(No)	Substantial out-of-pocket payments (Yes)	(No)	Adolescent mental health problems (Yes)	(No)	Low exclusive breastfeeding rates (Yes)	(No)	Aggressive promotion of unhealthy foods (Yes)	(No)	Increasing number overweight or obese (Yes)	(No)
% Overall	Yes, No	60	40	58	58	33	67	18	82	84	16	69	31	60	40	89	11
% EU14	Yes, No	57	43	43	57	21	79	14	86	79	21	29	71	50	50	86	14
% EU13	Yes, No	46	54	46	54	31	69	38	62	77	23	69	31	62	38	77	23
% CIS	Yes, No	70	30	70	30	50	50	0	100	80	20	50	50	50	50	80	20
% SEEHN	Yes, No	67	33	67	33	50	50	17	83	67	33	50	50	67	33	67	33

Schools are not a health promoting environment	Children are not taught in schools what they need to know about their health, present and future	Parents are not taught parenting skills	Late identification of children with developmental difficulties	Low or decreasing vaccination coverage	High rates of road and other injuries	Left-behind children whose parents working abroad	Increasing gambling and gaming among adolescents	Country
No	No	Yes	No	No	Yes	No	Yes	Albania
-	-	-	-	-	-	-	-	Andorra
Yes	No	Yes	Yes	Yes	Yes	No	Yes	Armenia
Yes	Yes	Yes	No	Yes	No	No	No	Austria
No	No	No	No	No	No	No	Yes	Azerbaijan
Yes	No	No	No	No	No	No	Yes	Belarus
No	Yes	No	No	Yes	Yes	No	Yes	Belgium
No	No	No	No	Yes	No	No	Yes	Bosnia and Herzegovina
No	No	No	No	No	No	Yes	Yes	Bulgaria
Yes	No	No	Yes	Yes	Yes	No	No	Croatia
No	No	Yes	Yes	No	Yes	No	No	Cyprus
No	No	No	No	Yes	No	No	Yes	Czechia
No	No	No	No	Yes	No	No	No	Denmark
Yes	Yes	Yes	Yes	Yes	Yes	No	No	Estonia
No	No	No	Yes	No	No	No	No	Finland
No	No	No	Yes	Yes	Yes	No	Yes	France
Yes	Yes	No	Yes	No	Yes	Yes	Yes	Georgia
No	No	No	No	Yes	No	No	No	Germany
Yes	Yes	No	No	Yes	Yes	No	No	Greece
Yes	Yes	Yes	Yes	No	Yes	No	Yes	Hungary
-	-	-	-	-	-	-	-	Iceland
No	No	No	No	No	No	No	No	Ireland
Yes	Yes	No	Yes	Yes	Yes	No	No	Israel
Yes	Yes	Yes	Yes	Yes	Yes	No	Yes	Italy
No	No	Yes	No	No	Yes	No	Yes	Kazakhstan
Yes	Yes	Yes	Yes	Yes	Yes	Yes	Yes	Kyrgyzstan
No	Yes	Yes	Yes	No	Yes	No	Yes	Latvia
Yes	Yes	Yes	Yes	Yes	Yes	Yes	Yes	Lithuania
Yes	No	No	Yes	Yes	Yes	No	No	Luxembourg
No	No	No	No	Yes	Yes	No	Yes	Malta
No	No	No	No	Yes	No	No	No	Monaco
-	-	-	-	-	-	-	-	Montenegro
Yes	No	No	No	Yes	No	No	No	Netherlands
-	-	-	-	-	-	-	-	North Macedonia
-	-	-	-	-	-	-	-	Norway
No	No	No	No	No	No	No	No	Poland
Yes	Yes	Yes	No	No	Yes	No	Yes	Portugal
Yes	Yes	Yes	Yes	Yes	Yes	Yes	No	Republic of Moldova
Yes	Yes	Yes	Yes	Yes	No	Yes	No	Romania
No	No	No	No	No	No	No	Yes	Russian Federation
-	-	-	-	-	-	-	-	San Marino
-	-	-	-	-	-	-	-	Serbia
No	No	No	No	Yes	No	No	Yes	Slovakia
No	No	No	No	No	No	No	No	Slovenia
No	No	No	No	No	No	No	Yes	Spain
No	No	No	No	No	No	No	No	Sweden
Yes	Yes	Yes	No	No	No	No	No	Switzerland
Yes	Yes	Yes	Yes	No	No	No	No	Tajikistan
Yes	No	No	No	Yes	Yes	No	No	Türkiye
No	No	No	No	No	No	No	No	Turkmenistan
-	-	-	-	-	-	-	-	Ukraine
No	No	No	No	No	No	No	No	United Kingdom
No	No	No	No	No	No	No	No	Uzbekistan

44	56	36	64	36	64	38	62	51	49	47	53	13	87	47	53	% Overall	Yes, No
43	57	36	64	21	79	29	71	64	36	43	57	0	100	36	64	% EU14	Yes, No
38	62	38	62	46	54	54	46	46	54	54	46	15	85	38	62	% EU13	Yes, No
50	50	30	70	50	50	40	60	30	70	40	60	20	80	60	40	% CIS	Yes, No
50	50	50	50	50	50	0	50	50	50	50	50	33	67	17	83	% SEEHN	Yes, No

Appendix S4. Early Childhood Development Framework

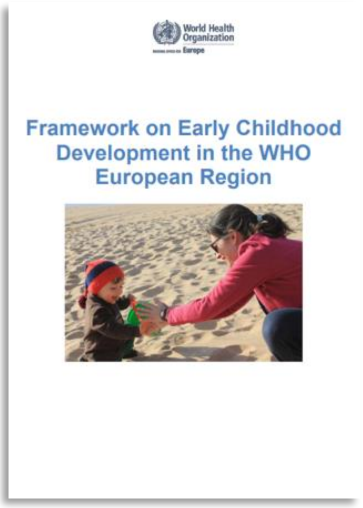

The European framework on Early childhood development aims to inform countries on measures they can take to enable young children to reach their full potential. It highlights three areas: (1) young children's needs, (2) monitoring a child's development and responding to developmental concerns, and (3) the social and environmental risks to development.
The document provides an adaptation for the European context of the global Nurturing Care Framework and gives additional guidance on how ECD can be promoted and how developmental difficulties can be prevented, identified and addressed within the diverse health systems in the Region.
The framework, an action-oriented policy document, is complemented by a tool to assess the situation of countries in this area.

Figure S2. ECD Framework in Europe

Link: https://apps.who.int/iris/handle/10665/332068

Appendix S5. Addressing the needs of primary care providers: a pocket book

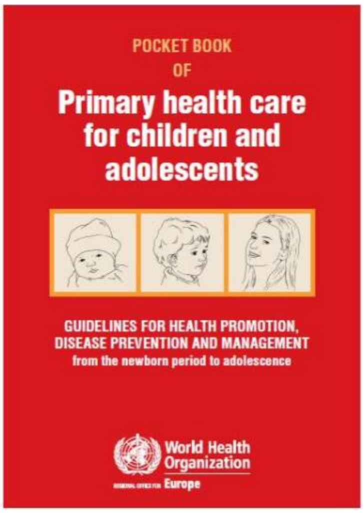

Figure S3.Pocket book for primary health care

The WHO *Pocket Book of Primary Health Care for Children and Adolescents* defines evidence-based standards of health care according to which every child attending primary health care services across the European Region should be cared for. It is organized around frequent complaints for which children seek care at the primary healthcare level and by causes of major mortality and morbidity. It includes a chapter that is specific to the newborn period and also one for adolescence. The chapter on well child visits and health promotion focuses on prevention. The Pocket Book is for use by doctors, nurses and other health workers who are responsible for the care of newborns, children, and adolescents at the PHC level and sets standards to which equipment and health care services should be available.

The *Pocket Book* aims to deliver on the promise of quality primary health care: its focus on evidence-based practices and prevention aims to counteract inappropriate medicalisation of health services (i.e. preferring invasive or active treatment over conservative or watchful management, intravenous treatment over oral rehydration therapy; and multiple drugs over just one) and prevent unnecessary treatment and hospitalization.

Link: https://apps.who.int/iris/handle/10665/352485

Appendix S6. Health Behaviour in School-aged Children (HBSC) study

For over 30 years the Health Behaviour in School-aged Children (HBSC) study, a WHO collaborative study, provides insights into adolescents' health behaviours and well-being. The study, undertaken every four years in 46 countries, representatively samples 11, 13 and 15 years old school children in Europe to get insights into their health-related knowledge and behaviours. The survey includes data on families, school, nutrition, physical activity, risk-taking and exploratory behaviours, and mental health among other areas. The shared questionnaire across countries enables comparison between countries, and since core questions remain over the years, trends can also be observed.

Country teams routinely prepare national reports and launch them with policy stakeholders, including adolescents, to discuss the findings and what should be done about them. This data strengthens regional data collection efforts and informs policy and practice nationally and regionally. The last study round took place in 2017/18. The 2017/2018 HBSC international report was launched in May 2020, together with a special issue of scientific papers.

Since the adoption of the European Child and Adolescent Health Strategy in 2015, eight new members (Azerbaijan, Cyprus, Georgia, Kazakhstan, Kyrgyzstan, Serbia, Tajikistan and Uzbekistan) have joined the study (see Figure 1). A new round is being undertaken in 2021/22, which will give insights into the impact of the COVID-19 pandemic on this age group.

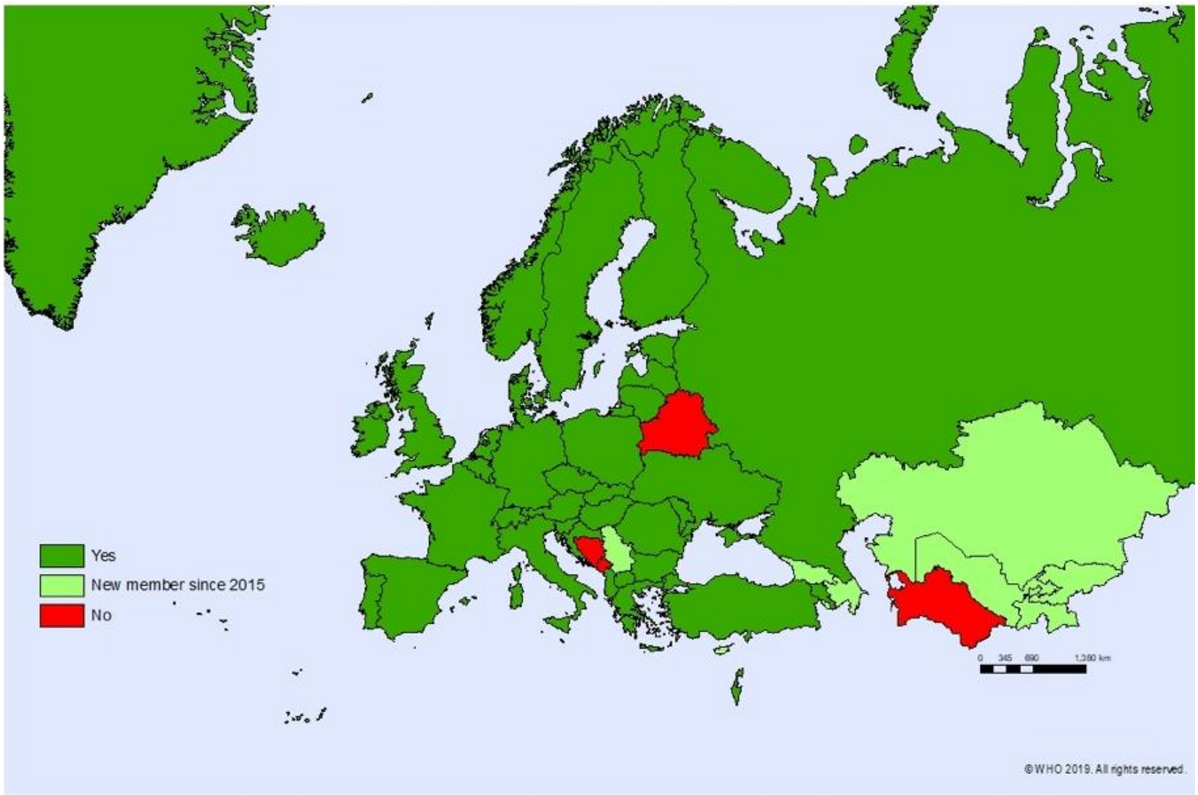

Figure S4. Member countries and regions of HBSC, 2020
